# Melatonin Improves Waterlogging Tolerance of *Malus baccata* (Linn.) Borkh. Seedlings by Maintaining Aerobic Respiration, Photosynthesis and ROS Migration

**DOI:** 10.3389/fpls.2017.00483

**Published:** 2017-04-05

**Authors:** Xiaodong Zheng, Jingzhe Zhou, Dun-Xian Tan, Na Wang, Lin Wang, Dongqian Shan, Jin Kong

**Affiliations:** ^1^College of Horticulture, China Agricultural UniversityBeijing, China; ^2^Beijing Soil and Fertilizer Work StationBeijing, China; ^3^Department of Cellular and Structural Biology, UT Health Science Center San Antonio, San AntonioTX, USA

**Keywords:** melatonin, waterlogging, ROS, oxidative stress, aerobic respiration, photosynthesis

## Abstract

Waterlogging, one of the notorious abiotic stressors, retards the growth of apple plants and reduces their production. Thus, it is an urgent agenda for scientists to identify the suitable remedies for this problem. In the current study, we found that melatonin significantly improved the tolerance of apple seedlings against waterlogging stress. This was indicated by the reduced chlorosis and wilting of the seedlings after melatonin applications either by leaf spray or root irrigation. The mechanisms involve in that melatonin functions to maintain aerobic respiration, preserves photosynthesis and reduces oxidative damage of the plants which are under waterlogging stress. Melatonin application also enhances the gene expression of its synthetic enzymes (MbT5H1, MbAANAT3, MbASMT9) and increases melatonin production. This is the first report of a positive feedback that exogenous melatonin application promotes the melatonin synthesis in plants. A post-transcriptional regulation apparently participated in this regulation. When exogenous melatonin meets the requirement of the plants it is found that the protein synthesis of MbASMT9 was suppressed. Taken together, the results showed that melatonin was an effective molecule to protect plant, particularly apple plant, against waterlogging stress.

## Introduction

Waterlogging, is a major agricultural constraint that limits crop growth and reduces their yield (Xu et al., 2013). It is frequently encountered during the raining seasons in many areas worldwide. The excessive waterlogging causes root damage, impairs the water uptake and, finally leads to chlorosis and wilting of the plants (Arbona et al., 2008). It was estimated that waterlogging stress resulted in nearly 40–80% of the crop yield loss in the area greater than 17 million km^2^ (Voesenek and Sasidharan, 2013; Shabala et al., 2014).

The reactive oxygen species (ROS) is believed to play a critical role in the response of plant to waterlogging stress. At the early stage during waterlogging, the elevated ROS molecules functions as an important second messenger in signaling for response. Following the prolonged waterlogging, the increased anaerobic respiration of root and the responsive stomata closure in leaves induce a burst of excessive ROS production. If the excessive ROS is not migrated properly, it will cause plant oxidative damage and finally, it leads to roots rotting and leaves wilting (Hossain et al., 2009).

Plants have already developed a series of antioxidant mechanisms to defend themselves against oxidative stress. These include small molecule antioxidants (SMA) and antioxidant enzymes (Apel and Hirt, 2004; Mittler et al., 2004). SMA includes ascorbic acid, carotenoids, tocopherol, glutathione, polyphenol, etc. They can scavenge ROS with different chemical reactions (Smirnoff, 2000; Kuzniak and Sklodowska, 2001; Foyer and Noctor, 2005). The antioxidant enzymes are mainly those of superoxide dismutase (SOD), catalase (CAT), peroxidase (POD), etc. Both SMA and antioxidant enzymes work coordinately to keep the oxidative stress in check (Hossain et al., 2009). In addition to the SMA mentioned above, melatonin (*N*-acetyl-5-methoxytryptamine) is a potent free radical scavenger and an antioxidant (Tan et al., 1993, 2012). Melatonin was identified in plants in Dubbels et al. (1995) and Hattori et al. (1995). Since then it has been reported to exist in many plants and plant products (Ramakrishna et al., 2012; Yip et al., 2013; Shi et al., 2015b; Zhao et al., 2015a; Li C. et al., 2016; Ma et al., 2016; Xu et al., 2016). Different from other antioxidants, it is an amphiphilic molecule which makes it distribute in all cellular compartments including cytosol, membrane, mitochondria and chloroplasts (López et al., 2009; Byeon et al., 2013; Back et al., 2016). Also, melatonin as well as its metabolites can eliminate different kinds of ROS including superoxide anion $\left(O_{2}^{•-}\right)$, hydrogen peroxide (H_2_O_2_), hydroxyl radical (^•^OH), singlet oxygen (^1^O_2_), peroxynitrite anion (ONOO^-^) and nitric oxide (NO) (Tan et al., 2013). Melatonin has been reported to protect plants against a variety of abiotic and biotic stresses (Zuo et al., 2014; Liang et al., 2015; Liu et al., 2015; Shi et al., 2015a; Wang et al., 2015; Zhao et al., 2015b; Li C. et al., 2016; Xu et al., 2016). The transgenic *Arabidopsis* plants, ecotopically expressing melatonin synthetic gene *MzASMT1*, had higher endogenous melatonin production and significantly lower ROS than that of their wild types. The melatonin enriched transgenic *Arabidopsis* had a greater tolerance to drought stress than the wild types (Zuo et al., 2014). The exogenous melatonin also improved tolerance of tomato plants against alkaline stress by migrating $O_{2}^{•-}$ and H_2_O_2_ (Liu et al., 2015). Melatonin application to apple leaves alleviated the drought-induced inhibition of photosynthesis (Wang et al., 2012). However, the effects of the exogenous melatonin application on apple plants which are under the sustained waterlogging condition have not been reported yet.

Reduced apple yield caused by waterlogging stress is a worldwide problem to be solved. Apple is a perennial woody plant, thus, the damage from waterlogging stress not only reduces apple yield of the current year, but also suppresses the tree vigor which also leads to the yield loss in the following years (Qu et al., 1999). *Malus baccata* (Linn.) Borkh. is used as a rootstock, which frequently suffers from the waterlogging stress (Wang et al., 2013). Therefore improvement of its waterlogging resistance by melatonin application will provide a potential cultivation method for apple production.

In current study, we investigated the protective effects of melatonin on *M. baccata* seedlings, which were subjected to the waterlogging stress. Furthermore, the potential mechanisms of these protections were also explored and discussed.

## Materials and Methods

### The Cultivation of Plant Material

Seeds of apple (*M. baccata*) were sown in soppy vermiculite. Two weeks later, the seedlings were watered with half-strength Hoagland’s nutrient solution (Li M.Q. et al., 2016). When the seedlings developed to have four leaves, they were watered with complete nutrient solution. The plants were kept in green house with the temperature at a constant 22 ± 2°C and a 16/8 h light/dark cycle. The light intensity was approximately 100 μmol⋅m^-2^⋅s^-1^.

### Waterlogging Stress/Melatonin Treatment and Sample Collection

After the *M. baccata* seedlings developed to have four leaves, a total of 96 seedlings were transplanted into the glass container with sterilized matrix soil. They were divided into eight groups. Plants in group I were watered with the 200 mL normal nutrient solution. Totally 25 mL normal nutrient solution was added every 3 days as control. The waterlogging stress was conducted in the remaining seven groups by keeping the soil being covered with 300 mL the nutrient solution and added 25 mL normal nutrient solution every 3 days.

The seedlings from group II was waterlogging stressed without supply of exogenous melatonin. The waterlogging stressed seedlings from group III to VIII were treated with different concentration of melatonin by spraying or irrigation. Melatonin was dissolved in 100% ethanol at a concentration of 10 mM and stored at -20°C as a stock solution. When use, melatonin was then diluted into 50, 100, and 200 μM, respectively with deionized water. These different concentrations of melatonin were sprayed to the leaves of seedlings every other day in group III, IV, and V, respectively. Two pieces of hardboard were used to avoid sprayed melatonin dropping into the soil. In group VI, VII, and VIII, melatonin was directly supplemented to the nutrient solution at the concentrations of 200, 400, and 600 μM, respectively. Groups I and II was also applied with equal volume of ethanol. The experiments were independently repeated three times. Photos were taken before and after 9 days of waterlogging stress/melatonin treatments.

The leaves and roots were collected from the all groups of seedlings, respectively, before and after 9 days of waterlogging stress/melatonin treatment.

### Melatonin Measurement

The leaves collected from the seedlings were immediately frozen at -80°C for future melatonin detection. Around 1 g of frozen leaves of *M. baccata* was ground to a fine powder in liquid nitrogen. The powder was mixed with 10 mL methanol and ultra-sonicated (80 Hz) for 35 min at 45°C. The sample preparation and HPLC detection of melatonin were performed as described by Zhao et al. (2013). Each experiment was independently repeated three times.

### RNA Extraction and RT-PCR Analysis

Total RNA was isolated from the leaves of seedlings of the eight groups, respectively, before and after 9 days of waterlogging stress/melatonin treatment, using the EASY spin Plant RNA Rapid Extraction Kit (Biomed, Beijing, China). The first-strand cDNA was synthesized following the protocol of Kit (Promega, Madison, WI, USA). The cDNAs were used as template for RT-PCR. The specific primers were designed according to the sequence of melatonin synthesized enzyme genes *MbASMT9* (KJ156531), *MbAANAT3* (KJ156532), and *MbT5H1*, respectively, by Primer 5 software and checked by BLAST search in the apple genome^1^ (*MbASMT9* Forward Primer 5′-TGATCTGCCCCATGTCGT-3′, Reverse Primer 5′-CTTTGTGGCGAGGGAAAC-3′; *MbAANAT3* Forward Primer 5′-CGCTCCCTAACTACCAACCA-3′, Reverse Primer 5′-ACAAATCCCTTTCCCTACCAG-3′; *MbT5H1* Forward Primer 5′-ATCCGTAAGATTTGTATACTTGAGCT-3′, Reverse Primer 5′-TCACCGACCAAGATAATAGCCT-3′).

RT-PCRs were performed use of 20 μL reaction mixtures containing 20 ng of first-strand cDNA, 2 × PCR Mix 5 μL (CWBIO, Beijing, China), 0.5 μM of each of the forward and reverse primers and appropriate amounts of ddH_2_O. The *Actin* gene was used as the internal standard, and the PCR program for *Actin* was as following: 94°C for 5 min; 28 cycles at 94°C for 30 s, 55°C for 30 s, and 72°C for 30 s; and 72°C for 10 min, amplified with primers (Forward Primer 5′-CAATGCCTGCCATGTATG-3′, Reverse Primer 5′-CCAGCAGCTTCCATTCCAAT-3′). The PCR products were analyzed in 1% TAE-agarose gel stained by goodview. The quantification of amplified *Actin*, *MbT5H1*, *MbAANAT3*, and *MbASMT9* fragment was done by the ImageJ software^2^, the ratios of *MbT5H1*, *MbAANAT3*, *MbASMT9* and *Actin* were calculated.

### The Cloning of *MbASMT9* Gene and the Expression of MbASMT9 Protein in *Escherichia coli*

The coding frame of *MbASMT9* was amplified and ligated into pMD 19-T Simple, which was then digested with BamHI/EcoRI and inserted into the pGEX-6p-1. The protein expression of MbASMT9 was analyzed according to Li et al. (2015). The purity of the GST-MbASMT9 protein was confirmed by SDS–PAGE.

### Protein Extraction and Western Blot Analysis

The total protein was isolated from leaves of seedlings according to Wang et al. (2006). Western blot was applied with antibody of MbASMT9 (rabbit, 1:3000). The recombinant GST-MbASMT9 was used as an antigen to raise polyclonal GST-MbASMT9 antibodies in rabbit. The preparation of MbASMT9 antibody was carried out in accordance with relevant guidelines and regulations. The experimental protocols of the MbASMT9 antibody preparation were reviewed and approved by Beijing Municipal Science and Technology Commission. The chemiluminescent signals were detected using an ECL detection kit (Amersham-Pharmacia, USA). The loading control of the Western blot was stained by Coomassie Brilliant Blue.

### Detection of Antioxidant Enzyme Activities

A total of 0.3 g leaves or 0.3 g roots of *M. baccata* seedlings were ground with 8 mL chilled 50 mM phosphate buffer (pH 7.8), then they were transferred into 10 mL tubes and centrifuged at 4°C for 15 min at 10,000 *g*. The supernatants were diluted with phosphate buffer to 10 mL and used for enzyme activity detection. The enzyme activity of SOD was detected according to the method of Stewart and Bewley (1980). CAT activity was measured as the absorbance at 240 nm wave length according to the method of Ma et al. (2016). POD (Peroxidase) activity was measured by the changes in absorbance at 470 nm due to guaiacol oxidation according to the method of Shi et al. (2015b). Each experiment was independently repeated at least three times.

### Measurements of Enzyme Activities of Alcohol Dehydrogenase (ADH) and Succinate Dehydrogenase (SDH)

A total of 0.3 g roots for each group were collected to detect the enzyme activity of ADH and SDH before and after 9 days of waterlogging stress/melatonin treatment to identify the alterations of anaerobic and aerobic respiration. ADH activity was measured as reported by Yamashita et al. (2015). SDH activity was detected according to Landi et al. (2009). Each experiment was independently repeated at least three times.

### Detections of ROS Level and Malondialdehyde (MDA) Content

The ROS level in roots was detected according to the method modified from Zuo et al. (2014). The intact roots were collected from each group and the fresh roots were immediately used for ROS detection before and after 9 days of waterlogging stress/melatonin treatment. Simply, the entire roots were incubated with the 5-(and 6)-chloromethyl-2′-7′-dichlorodihydrofluorescein diacetate acetyl ester (CM-H_2_DCFDA) solution for 20 min and washed with distilled H_2_O to remove excess CM-H_2_DCFDA. The fluorescence images were obtained with a Leica stereoscope (Leica, Wetzlar, Germany) (× 10).

In addition to roots, the ROS level in leaves was also detected. The leaves were collected from the each group, respectively before and after 9 days of waterlogging stress/melatonin treatments and used for detection of $O_{2}^{•-}$ and H_2_O_2_ immediately. For $O_{2}^{•-}$ measurement, the leaf histochemical staining was vacuum infiltrated with 0.1 mgmL^-1^ nitroblue tetrazolium in 25 mM K-HEPES buffer (pH 7.9) for 40 min. Then the samples were kept at 25°C in dark for an additional 4 h. For the H_2_O_2_ detection the leaves were vacuum infiltrated with 0.1 mg⋅mL^-1^ DAB in 50 mM Tris-acetate (pH 3.8) and were incubated at 25°C in dark for 24 h. Then leaves for either $O_{2}^{•-}$ or H_2_O_2_ detection were washed in 80% ethanol every 10 min at 80°C until the leaves lost green color completely (Gong et al., 2014). The MDA detection in the leaf samples were performed according to Zhao et al. (2013). Each experiment was independently repeated at least three times.

### Analyses of Chlorophyll (Chl) Content and Photosynthetic Rate (Pn) in Leaves of Seedlings

The extraction of Chl was conducted according to Porra et al. (1989). A total of 0.2 g leaves were homogenized in 2–3 mL 80% acetone. After filtration with four layers of gauze (1 mm × 1 mm), the homogenate was diluted with 80% acetone to 25 mL. The absorbance of the extract was measured at 645 and 663 nm. Chl concentration was calculated from the following equations: Chl a = 12.72 × OD663 -2.59 × OD645; Chl b = 22.88 × OD645 -4.67 × OD663; chl = chl a + chl b (Arnao and Hernández-Ruiz, 2009).

The photosynthetic rate was measured by LI-6400XT (LI-COR, Lincoln, NE, USA) according to the producer’s protocol. The light intensity was at 800 μmol⋅m^-2^⋅s^-1^. The humidity was about 50% and the temperature was 23°C. Each experiment was independently repeated three times.

### Statistical Analysis

The data are expressed as means ± SD. One-way ANOVA was used for the normality evaluation followed by a Tukey–Kramer multiple comparison test. The statistical significant difference was set up when *P* < 0.05. Statistical evaluations were carried out using SPSS software (IBM, Armonk, NY, USA).

## Results

### Melatonin Applications Improved the Tolerance of *M. baccata* Seedlings against Waterlogging Stress

**FIGURE 1:**
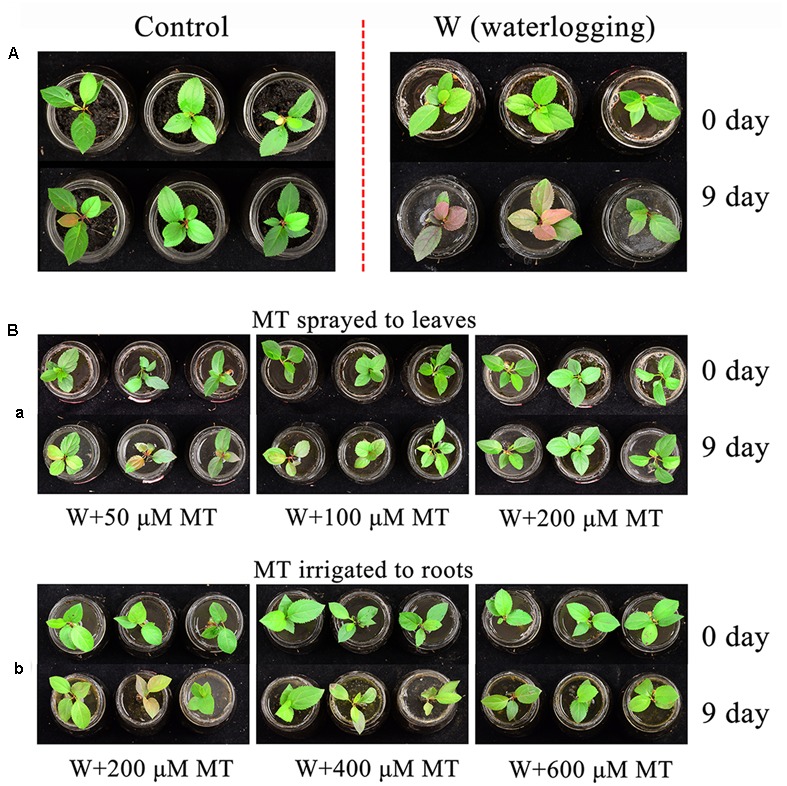
**The effects of melatonin on the *M. baccata* seedlings which were suffered from waterlogging stress. (A)** Before and after waterlogging stress. **(B)**: (a) The phenotype of *M. baccata* seedlings with melatonin leaf spray at different concentrations before and after waterlogging stress. (b) The phenotype of *M. baccata* seedlings with melatonin irrigation at different concentrations before and after waterlogging stress.

The results showed that waterlogging stress significantly impaired the *M. baccata* seedling growth. Those plants which were under waterlogging stress for 9 days wilted severely compared to the normal controls (**Figure 1A**). When melatonin was sprayed to the seedlings at the concentrations of 50, 100, and 200 μM, respectively, it was apparent that melatonin spraying improved tolerance of seedlings against waterlogging stress. The protective effects of melatonin indicated a dose-responsive manner. The seedlings treated with 200 μM of melatonin, even under the waterlogging stress, showed a similar phenotype as the normal controls. The similar results were also observed in the seedlings those melatonin was irrigated (**Figure 1B**). When melatonin was irrigated to the seedlings at the concentrations of 200, 400, and 600 μM, respectively, it also increased waterlogging resistance of seedlings as a dose-dependent manner. The irrigation with 600 μM of melatonin, gained a similar phenotype as spraying with 200 μM melatonin. The percentage of leaf chlorosis was also analyzed. It was clearly to see that the percentage of leaf chlorosis was significantly increased after waterlogging treatment. After melatonin was applied to the seedlings under waterlogging, the percentage of leaf chlorosis were significantly decreased (Supplementary Figure 1).

### The Effects of Waterlogging Stress and Melatonin Application on *De novo* Melatonin Synthesis in Seedlings

**FIGURE 2:**
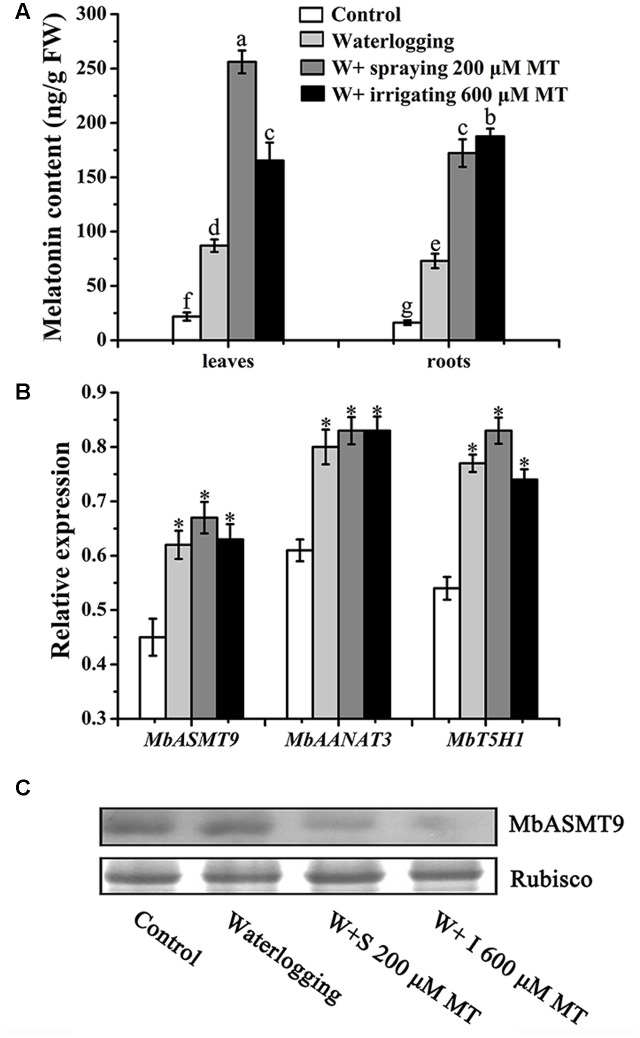
**Effects of waterlogging and exogenous melatonin application on the melatonin synthesis in *M. baccata* seedlings 9 days after waterlogging stress. (A)** Endogenous melatonin levels in leaves and in roots of the *M. baccata* seedlings. **(B)** The relative expression of melatonin synthetic genes (*MbASMT9*, *MbAANAT3* and *MbT5H1*) in the leaves. **(C)** The protein level of MbASMT9 detected by Western blot analysis in the leaves. The data are means ± SD of triplicate experiments. Asterisks (^∗^) and different letters indicate significant differences from the control (*P* < 0.05).

To find the potential effects of waterlogging stress and melatonin application on the endogenous melatonin synthesis, the melatonin level and its synthetic gene expression were detected. The results showed that waterlogging stress significantly elevated plants melatonin levels in leaves (87 vs. 21 ng/g FW of control) and in roots (73 vs. 16 ng/g FW of control) (**Figure 2A**).

When exogenous melatonin was applied to the stressed seedlings, their endogenous melatonin was further increased compared to the plants under waterlogging stress alone (**Figure 2A**). For example, the endogenous leaf melatonin level in melatonin (200 μM) sprayed plants was 256 ng/g FW, which was 2.9 times higher than that in leaves of waterlogged seedling alone (87 ng/g FW). The similar results were observed in roots and also in melatonin irrigated seedlings. In accordance with the elevated melatonin production, the gene expressions of melatonin synthetic enzymes including ASMT (acetylserotonin *O*-methyltransferase), AANAT (aralkylamine *N*-acetyltransferase) and T5H (tryptamine 5-hydroxylase), were slightly upregulated under the waterlogging stress and melatonin treatment compared to the controls (**Figure 2B**). The upregulated gene expression of *ASMT9*, which is the supposed melatonin synthetic rate-limiting enzyme, failed to result in the increase in its protein level. In contrast, its protein level was declined when compared to the controls (**Figure 2C**). Obviously, the post-transcriptional regulation occurred for *ASMT9.*

### The Effects of Melatonin on Antioxidant Enzymes

**FIGURE 3:**
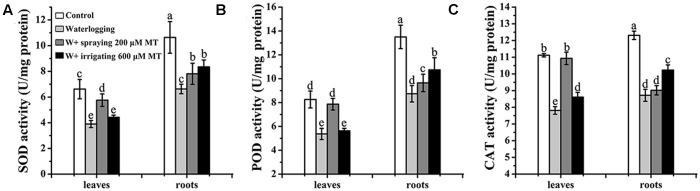
**Effects of melatonin on activities of antioxidant enzymes. (A)** Superoxide dismutase (SOD). **(B)** Peroxidase (POD). **(C)** Catalase (CAT) in both leaves and roots. The waterlogging stress was lasted 9 days. The data are means ± SD of triplicate experiments. Different letters indicate significant differences from the control (*P* < 0.05).

The activities of the antioxidant enzymes including SOD, POD, CAT were measured in four groups of *M. baccata* seedlings. These groups included the normal control group, waterlogging stress alone, waterlogging stress treated with melatonin (200 μM spray and waterlogging stress treated with melatonin (600 μM) irrigation. Waterlogging stress significantly suppressed all antioxidant enzymes tested in both levels and roots of the seedlings. However, melatonin treatment could recover the activities of these antioxidant enzymes suppressed by waterlogging stress in a great degree. For example, melatonin (200 μM) spray recovered the activity of CAT in leaves to the level comparable to the control seedlings (**Figure 3**).

### Effects of Melatonin on Aerobic and Anaerobic Respiration of *M. baccata* Seedlings under Waterlogging Stress

**FIGURE 4:**
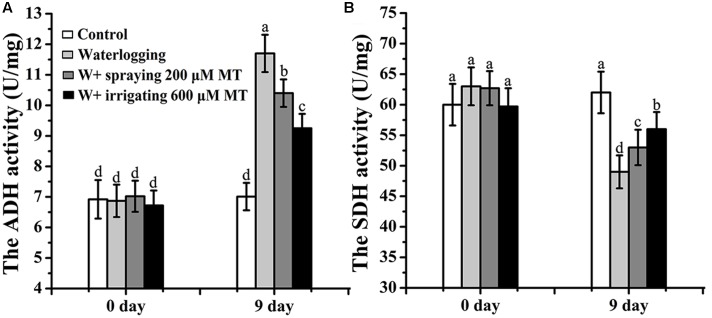
**Effects of melatonin on aerobic and anaerobic respirations of *M. baccata* seedlings under waterlogging stress. (A)** Anaerobic respiration which is indicated by ADH activity. **(B)** Aerobic respiration which is indicated by SDH activity. The waterlogging stress was last for 9 days. The data are means ± SD of triplicate experiments. Different letters indicate significant differences from the control (*P* < 0.05).

The activities of ADH and SDH indicate the anaerobic and aerobic respirations, respectively. It was found that the ADH activity (11.71 U/mg), the index of anaerobic respiration, was significantly higher in the roots of waterlogging stressed seedlings than that of normal control (7.01 U/mg). Both melatonin spray and irrigation reduced the anaerobic respiration indicated by decreased ADH activity (**Figure 4A**). In contrast, waterlogging stress suppressed the aerobic respiration indicated by the significantly reduced SDH activity compared to the control seedlings. This tendency was reversed by melatonin treatment in a great degree (**Figure 4B**).

### The Effect of Melatonin on $O_{2}^{•-}$ and H_2_O_2_ Production and the Oxidative Damage Induced by Waterlogging Stress

**FIGURE 5:**
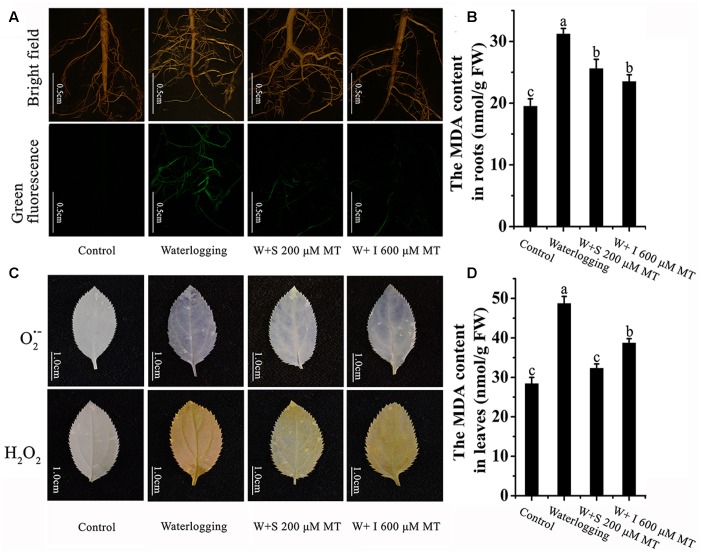
**Effects of melatonin on ROS levels and oxidative damage in *M. baccata* seedlings which were under waterlogging stress. (A)** ROS level in roots. **(B)** MDA production in roots. **(C)** The levels of superoxide anion and hydrogen peroxide in leaves. **(D)** MDA production in leaves. The waterlogging stress lasted for 9 days. Scale bars in **(A)** represent 0.5 cm. Scale bars in **(C)** represent 1cm. The data are means ± SD of triplicate experiments. Different letters indicate significant differences from the control (*P* < 0.05).

Waterlogging stress significantly elevated the $O_{2}^{•-}$ and H_2_O_2_ production both in leaves and in roots of the seedlings. These were indicated by the increased florescent staining intensities on them. Melatonin treatment significantly reduced $O_{2}^{•-}$ and H_2_O_2_ productions, no matter melatonin was sprayed to the leaves or irrigated to the roots of the seedlings (**Figures 5A,C**). Accordantly, waterlogging stress caused oxidative damage in both leaves and roots of the seedlings. This was indicated by the increased content of MDA. It was expected that melatonin treatment significantly reduced the MDA levels in leaves and also in roots (**Figures 5B,D**).

### The Effect of Melatonin on the Chlorophyll Content and Photosynthetic Rate

**FIGURE 6:**
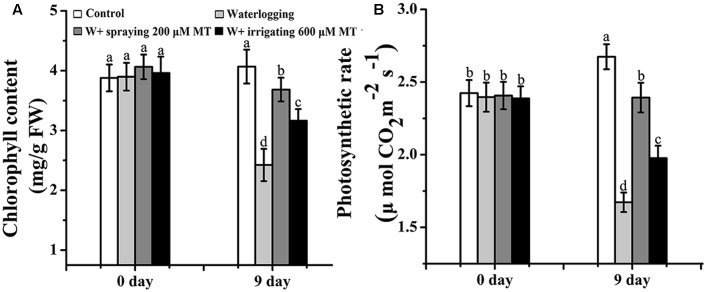
**Effects of melatonin on the chlorophyll content and photosynthetic rate of the *M. baccata* seedlings before and after waterlogging stress. (A)** Chlorophyll content. **(B)** Photosynthetic rate. The data are means ± SD of triplicate experiments. Different letters indicate significant differences from the control (*P* < 0.05).

Waterlogging stress dramatically reduced the Chl content in the leaves of seedlings. After 9 days of waterlogging stress, the Chl content of these plants (2.4 mg/g FW) was only roughly half of the value of control seedlings (4.1 mg/g FW). The photosynthetic rate of the waterlogging stressed plants was 1.67 μmol⋅m^-2^⋅s^-1^. The value was significantly lower than that of control seedlings (2.67 μmol⋅m^-2^⋅s^-1^). Melatonin treatments (spray or irrigation) improved both Chl content and photosynthetic rate which were suppressed by the waterlogging stress (**Figures 6A,B**).

## Discussion

Waterlogging, caused by poor drainage, flooding, and long periods of rainfall, usually occurs in summer and autumn, which hampers apple tree growth and results in yield loss (Yu et al., 2015). Because of the global warming effect, it is predicted that this climate change will bring more rainfall yearly and thus the waterlogging will be frequently encountered by the crops worldwide. This will bring severe problem for these crops which are intolerant to the waterlogging stress including the apple trees. To solve this problem, it has become an urgent agenda for scientist.

In the current study, a unique molecule, melatonin, was tested with this purpose. Melatonin was reported to enhance plant tolerance against various abiotic stressors including cold, hot, drought, salinity, and chemical pollutants (Bajwa et al., 2014; Wei et al., 2014; Liang et al., 2015; Liu et al., 2015; Shi et al., 2015b; Li X. et al., 2016; Xu et al., 2016). But the effect of melatonin on waterlogging resistance is still to be investigated.

Long lasting waterlogging caused apple seedling damage. Under waterlogging, the seedlings changed their metabolism from aerobic to anaerobic respiration (Fukao and Bailey-Serres, 2004). During this transition, a burst of ROS was generated and this resulted in oxidative damage of the seedlings (Hossain et al., 2009; Xu et al., 2013; Takahashi et al., 2015). This was indicated by the phenotype alterations of the seedlings such as chlorosis and wilting (**Figure 1**). However, all these changes were partially or completely prevented by melatonin application either by spraying to the leaves or irrigating to the roots. The primary mechanism of melatonin to improve the tolerance of apple seedlings against waterlogging stress may relate its potent free radical scavenging and antioxidant capacity (Tan et al., 1993, 2012). Melatonin not only directly scavenges the ROS but also upregulates the activities of a variety of antioxidant enzymes (Wang et al., 2012). This was in accordance with our observations in the current study (**Figures 3**, **5**).

In the current study, both waterlogging stress and melatonin application significantly increased melatonin level of the plants. It is well known that various stressors induce melatonin production in plants as well as in animals (Byeon et al., 2012; Li C. et al., 2016; Xu et al., 2016). This is considered as self-defense of organisms against external insults (Tan et al., 2015). Our observation provided new evidence to support this consideration. Waterlogging induced the gene expression of the melatonin synthetases and increased melatonin production in the apple seedlings. This positive response mechanism at the RNA level, for melatonin production in apple plants, probably also, exists in other plants. When exogenous melatonin was applied under waterlogging stress, the stress-induced expression of the melatonin synthetases still maintained their high expression. But obviously, the slight expression change between melatonin + waterlogging treatment and only waterlogging treatment can’t explain the significant melatonin increase after exogenous melatonin applied under waterlogging. It seemed that melatonin absorbed from outside mainly resulted in melatonin increase. In addition, for the MbASMT9, a melatonin synthetic rate-limiting enzyme, which was located in chloroplasts (Kang et al., 2013; Zheng et al., 2017), its upregulated RNA expression did not result in elevated protein level. In contrast, its protein level reduced after melatonin application. This probably was the first report to document a post-transcriptional regulation of melatonin synthesis in plants. There are accumulated post-transcriptional regulation reported, including RNA processing, transport and degradation, translation control to fine-tune biological processes for plants in response to the environmental changes (Palatnik et al., 2003, 2007; Cowley et al., 2012; Saze et al., 2013). Obviously, herein, the post-transcriptional regulation conserves resources and energy of the plants to avoid extra melatonin production when exogenous applied melatonin meets their requirement. This observation provided valuable information as to use of exogenous melatonin to improve plant tolerance to against stressors.

**FIGURE 7:**
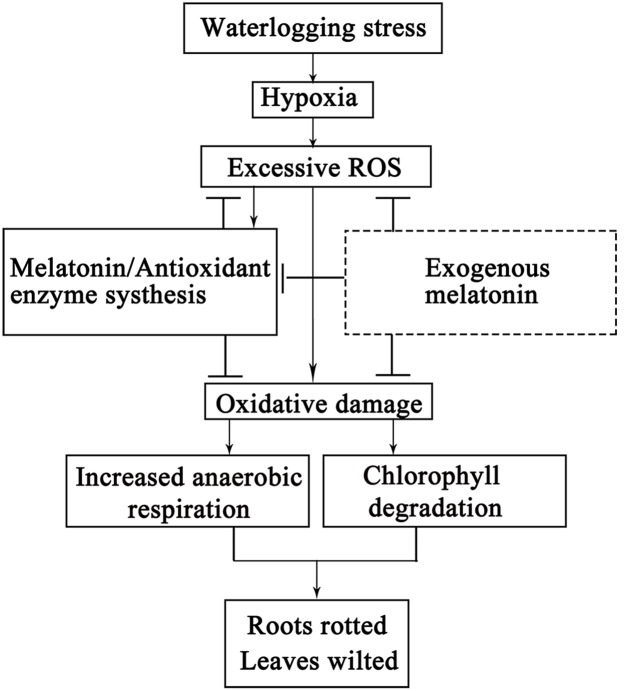
**The diagraph of the speculated mechanisms regarding the oxidative damage induced by waterlogging stress and the protective effects of melatonin application**. Arrow heads indicated the stimulation and the even bars indicated the inhibition.

There are accumulated reports described the waterlogging caused transition from aerobic to anaerobic respiration in root, which is also confirmed by us (**Figure 4**). Our results also uncovered the role of melatonin to maintain aerobic respiration under waterlogging stress, by efficient suppression of the ROS burst and subsequent mitochondria degradation. The chlorosis is a typical sign unavoidably happening after severe waterlogging. Due to the reason that waterlogging leads to water shortage and subsequent quick stomata closure, the high concentration of O_2_ can’t be released out and photosynthetic electron transportation is blocked in chloroplasts. Therefore, the ion leakage from the electron transport chain would induce in over-produced $O_{2}^{•-}$ and H_2_O_2_, which destroy chlorophyll and lead to the disintegration of chloroplasts (Musgrave, 1994; Smethurst and Shabala, 2003). The protective effects of melatonin on Chl decay, photosynthetic capacity and stomata configurations have been reported previously in other stressors such as in drought and hot (Wang et al., 2012; Xu et al., 2016). Actually, chloroplasts, as one of the most suffered organelles from ROS, were proved to be the major site for melatonin production. A large amount of melatonin is needed to maintain its structure and function. Therefore absorbed and *in vivo* synthesized melatonin can function together to migrate waterlogging induced ROS and help to survive the stress. Here we reported that melatonin application preserved the Chl content and maintained the photosynthetic rate in seedlings suffered from waterlogging stress. The high content of Chl and efficient photosynthesis are required for high yield of apple production (Li et al., 2000). It is our speculation that melatonin application in the field will increase the tolerance of apple tree and reduces apple yield loss against waterlogging stress. The speculated mechanisms are summarized in **Figure 7**.

## Author Contributions

Designed the studies: JK, Undertook the experimental work: XZ, JZ, NW, LW, and DS. Contributed to figures and manuscript preparation: XZ, D-XT, and JK. All authors read and approved the final manuscript.

## Conflict of Interest Statement

The authors declare that the research was conducted in the absence of any commercial or financial relationships that could be construed as a potential conflict of interest.

## Footnotes

1 https://www.rosaceae.org/species/malus/all

2 http://en.wikipedia.org/wiki/ImageJ
