# Supplementary material for: Melatonin Improves Waterlogging Tolerance of Malus baccata (Linn.) Borkh. Seedlings by Maintaining Aerobic Respiration, Photosynthesis and ROS Migration
Source: Front Plant Sci. 2017 Apr 5;8:483. doi: 10.3389/fpls.2017.00483 (PMC5380759; doi:10.3389/fpls.2017.00483)
Supplement: Supplementary file 1 [file Presentation_1.PDF]

Supplementary Material

Melatonin Improves Waterlogging Tolerance of *Malus baccata* (Linn.) Borkh. Seedlings by Maintaining Aerobic Respiration, Photosynthesis and ROS Migration

Xiaodong Zheng*, Jingzhe Zhou, Dun-Xian Tan, Na Wang, Lin Wang, Dongqian Shan, Jin Kong

*** Correspondence: Jin Kong**: jinkong@cau.edu.cn

1. Supplementary Figures

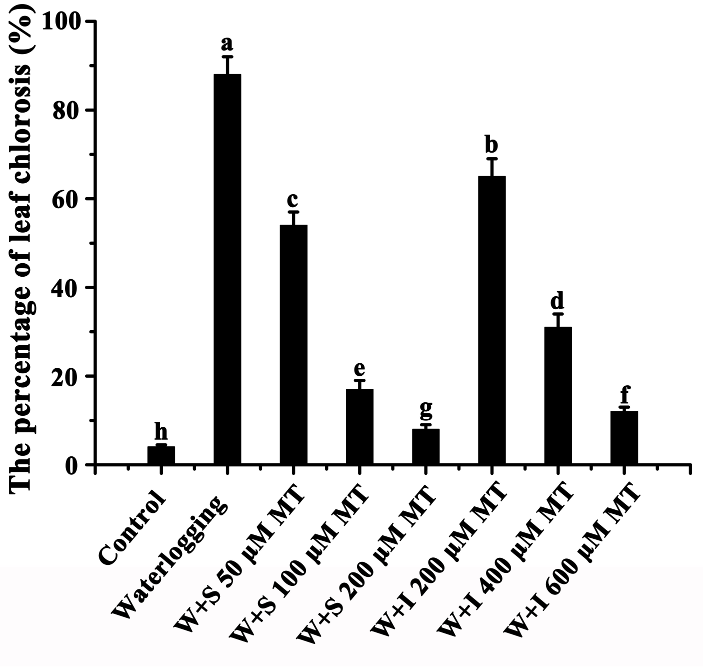

Supplementary Figure 1. The effects of melatonin application on the percentage of leaf chlorosis of *M. baccata* seedlings after 9 days' waterlogging treatment. The data are means ± SD of triplicate experiments. Different letters indicate significant differences from the control ($P < 0.05$).
